# Supplementary material for: TSG-6+ cancer-associated fibroblasts modulate myeloid cell responses and impair anti-tumor response to immune checkpoint therapy in pancreatic cancer
Source: Nat Commun. 2024 Jul 10;15:5291. doi: 10.1038/s41467-024-49189-x (PMC11237123; doi:10.1038/s41467-024-49189-x)
Supplement: Supplementary file 1 — Supplementary Information [file 41467_2024_49189_MOESM1_ESM.pdf]

TSG-6+ Cancer-Associated Fibroblasts Modulate Myeloid Cell Responses and Impair Anti-Tumor Response to Immune Checkpoint Therapy in Pancreatic Cancer

Supplementary Figure 1

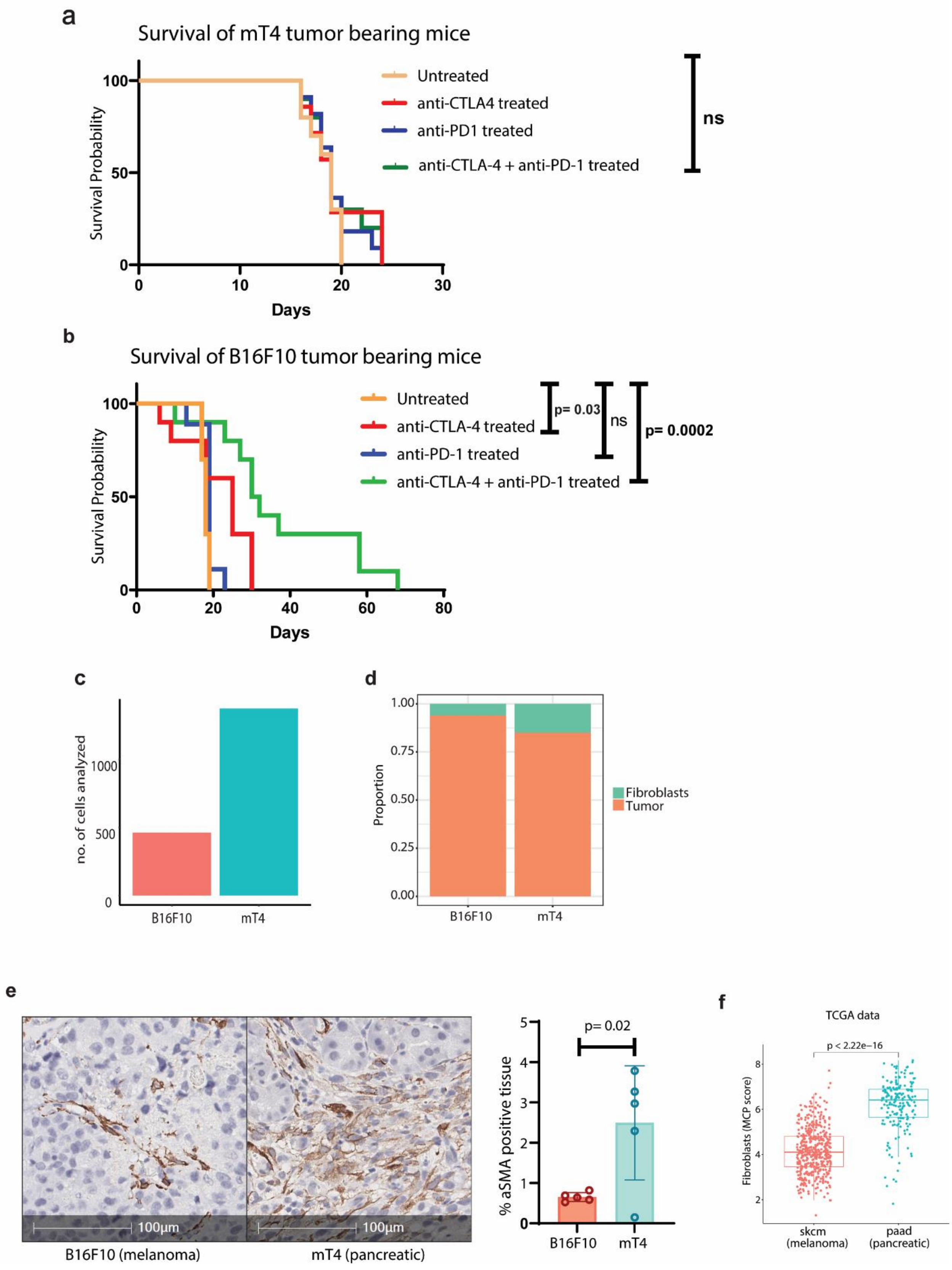

Supplementary Figure. 1: Fibroblast abundance is higher in ICT-resistant pancreatic tumors compared to ICT-sensitive melanoma tumors. a) Kaplan-Meier survival analysis of B16F10 tumor bearing mice randomized into the four treatment groups (n= 10 mice in each group). b) Kaplan-Meier survival analysis of mT4 tumor bearing mice randomized into the four treatment groups (n= 10 mice in each group). Data representative of two independent experiments. Statistical significance was calculated using Log-rank Mantel-Cox test (two-sided). p values for each comparison has been indicated in the figure. ns, non-significant. c) Box plot representing the total number of cells analyzed in the scRNAseq experiment for each tumor type (pooled sample from 3 mice). d) Plot depicting abundance of fibroblasts in proportion to total recovered cell counts in B16F10 and mT4 tumors. e) Representative immunohistochemistry staining images and quantification of alpha smooth muscle actin (aSMA) positive staining in B16F10 (n= 5 mice) and mT4 tumors (n= 5 mice). Scale bar = 100µm. Statistical significance was calculated using Student's t-test (two-tailed). Data are presented as mean values +/- SD. f) Microenvironment Cell Populations (MCP)- scoring of fibroblast abundance in The Cancer Genome Atlas (TCGA) dataset of patients with melanoma (skcm, skin cutaneous melanoma) (n= 480 patients) and pancreatic cancer (paad, pancreatic adenocarcinoma) (n= 186 patients). Data are presented as mean values +/- SD. Statistical significance was calculated using Student's t-test (two-tailed) and p values for each comparison has been indicated in the figure. The center of the plot represents mean of the group and the whiskers represent minimum- maximum values. Source data are provided as a Source Data file.

Supplementary Figure 2

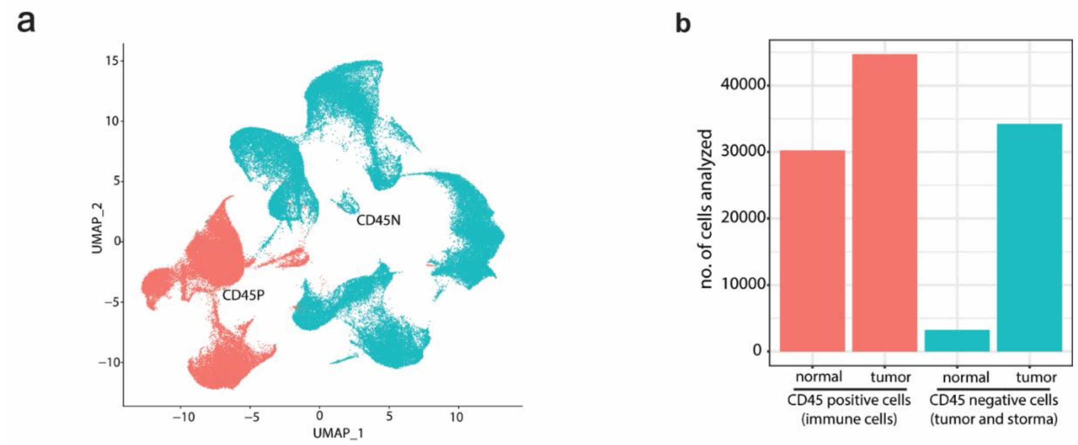

Supplementary Figure 2: scRNAseq analysis of human PDAC tumors. a) UMAP plot depicting total number of CD45 positive highlighted in red (CD45P; immune cells) and CD45 negative highlighted in blue (CD45N; non-immune cells) reanalyzed from Peng *et al*[1]. b) Quantification of total number of cells analyzed from each group depicted in a) (n = 24 PDAC patients and 11 normal pancreas).

Supplementary Figure 3

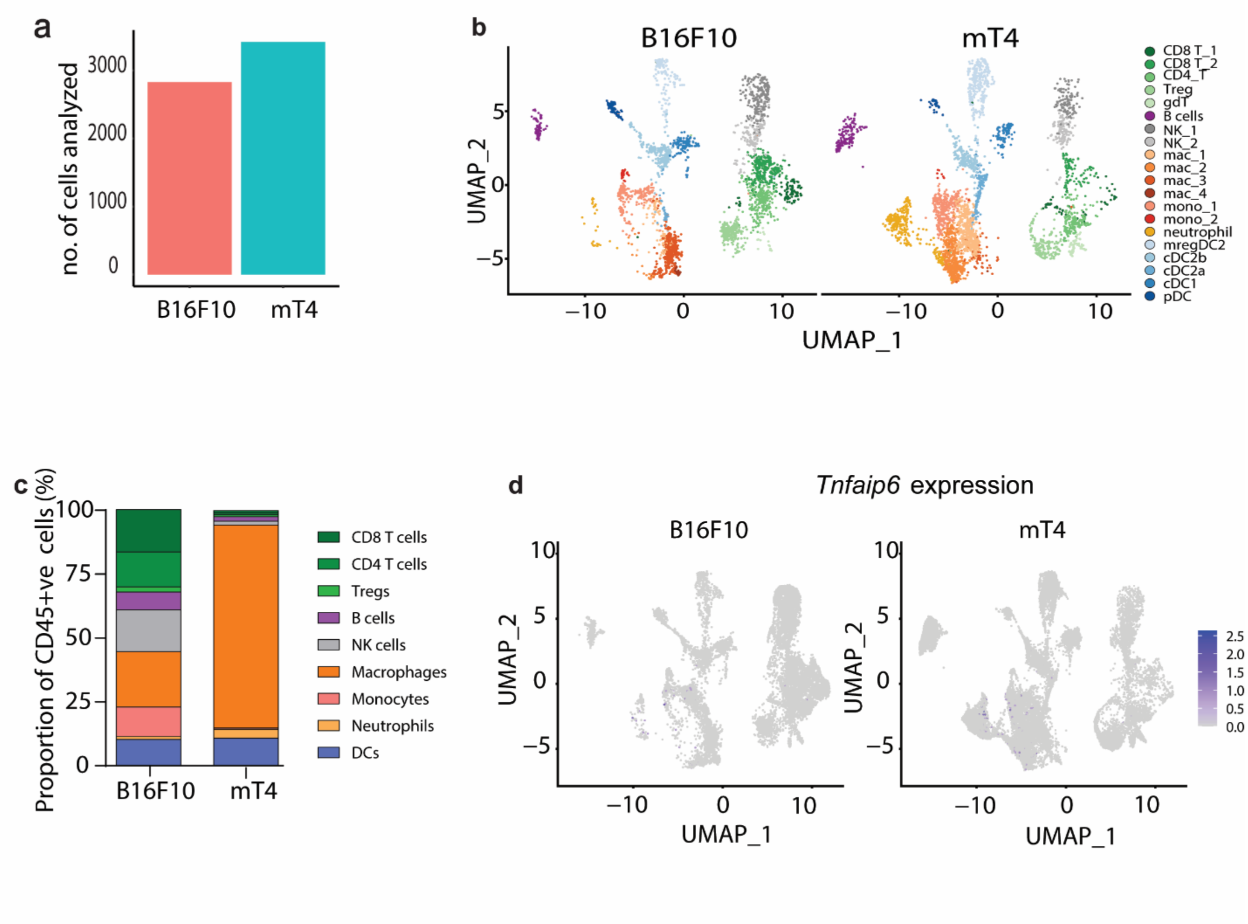

Supplementary Figure 3: Intratumoral immune cell analysis of murine melanoma and pancreatic tumors using scRNAseq and CyTOF. a) Box plot depicting total number of cells obtained and assessed in the CD45 positive murine scRNAseq dataset (pooled sample from 3 mice). b) UMAP plot represented in Figure. 3b split by the tumor type to indicate distribution of cell clusters. c) Relative frequencies of immune cell populations identified via CyTOF analysis as a proportion of total CD45+ cells performed on murine mT4 pancreatic tumors (n= 6 mice) and B16F10 melanoma tumors (n= 5 mice). Based on the markers used, the populations were defined as CD8 T cells (CD8+ CD3e+), CD4 T cells (CD4+ CD3e+), Tregs cells (FoxP3+ CD4+ CD3e+), B cells (CD19+), NK cells (NK1.1+), macrophages (CD11b+ F4/80+), monocytes (CD11b+Ly6c+F4/80-), neutrophils (CD11b+Ly6C+Ly6G+) and dendritic cells (DCs)

(CD11c+MHCII+). d) UMAP plot representing *Tnfaip6* expression in all immune cells analyzed from scRNAseq performed on B16F10 and mT4 tumors. Source data are provided as a Source Data file.

Supplementary Figure 4

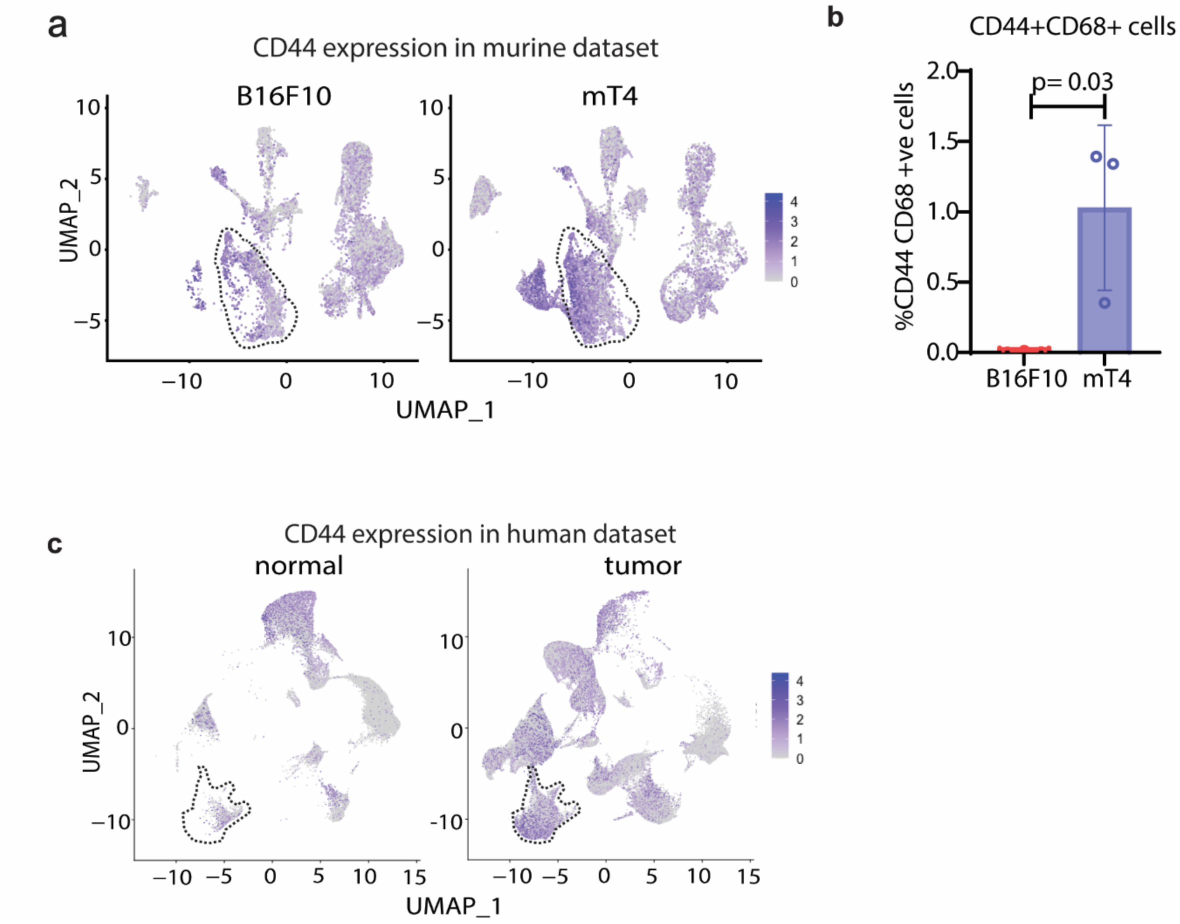

Supplementary Figure 4: CD44+ macrophages are enriched in pancreatic tumors. a) UMAP plot depicting *Cd44* expression in all immune cells analyzed from scRNAseq performed on B16F10 and mT4 tumors. Enclosed area in black dotted line highlights the macrophage clusters. b) Quantification of frequency of CD44+CD68+ cells in B16F10 (n= 3 mice) and mT4 tumor tissues (n= 3 mice). Data are presented as mean values +/- SD. Statistical significance was calculated using Student's t-test (two-tailed). p values for each comparison has been indicated in the figure. Source data are provided as a Source Data file. c) UMAP plot depicting *CD44* expression in all cells in human PDAC dataset reanalyzed from Peng *et al*[1]. Enclosed area in black dotted line highlights the myeloid clusters.

Supplementary Figure 5

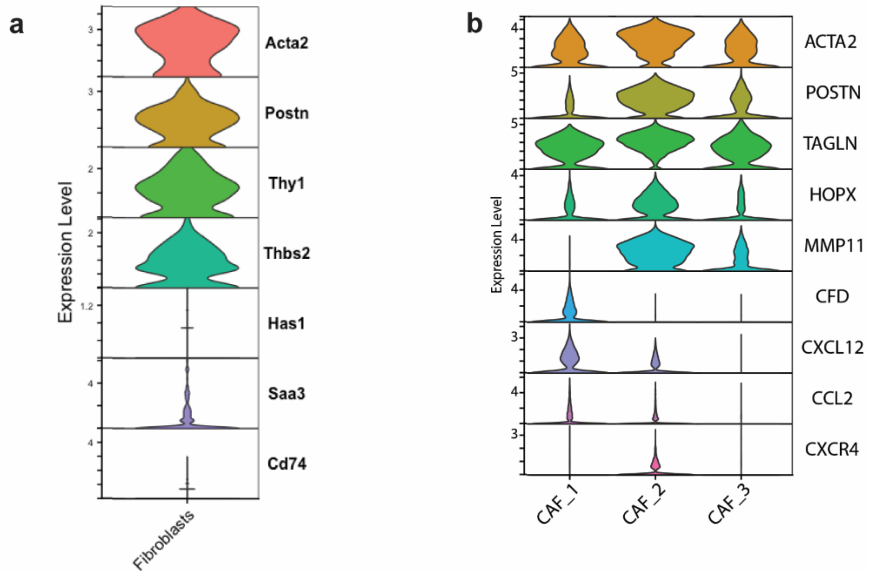

Supplementary Figure 5: Cancer-associated fibroblasts present in pancreatic tumors predominantly express myCAF-like phenotype. a) Violin plots depicting expression of genes used to characterize cancer associated fibroblast (CAF) subsets in the a) murine and b) human dataset[1], as classified by Elyada *et al*[2] (n = 24 PDAC patients and 11 normal pancreas).

Supplementary Figure 6

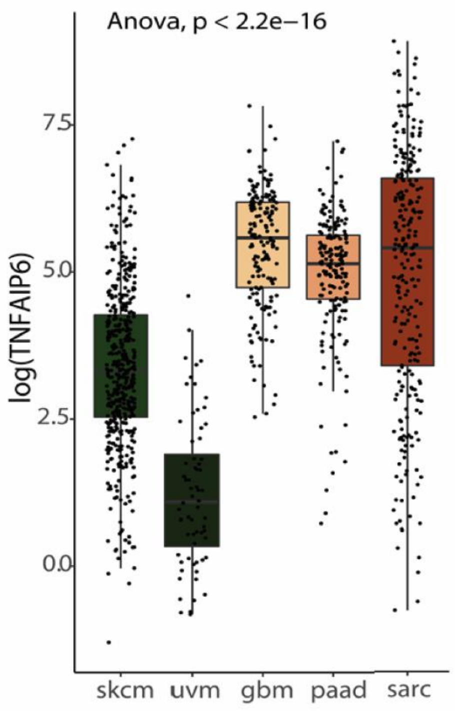

Supplementary Figure. 6: Elevated TSG-6 gene expression is observed in other ICT-resistant tumor types. *TNFAIP6* expression in The Cancer Genome Atlas (TCGA) dataset in immune checkpoint therapy (ICT) more-responsive tumor types (green shades) and less-responsive tumor types (brown shades). Each dot represents a patient. Statistical significance was calculated using one-way ANOVA and p value for the comparison has been indicated in the figure. Data are presented as mean values +/- SD. The center of the plot represents mean of the group and the whiskers represent minimum- maximum values. skcm, skin cutaneous melanoma (n= 480); uvm, uveal melanoma (n= 80); gbm, glioblastoma (n= 619); paad, pancreatic adenocarcinoma (n= 186); sarc, sarcoma (n= 265).

Supplementary Table 1

Pancreatic Cancer

Sample name	Sex	Clinical staging	Treatment at time of resection	Analysis performed
A	F	IIB	Untreated	TSG-6 expression/ infiltration analysis
B (location 1)	F	IIB	Untreated	Infiltration analysis
C	F	III	Untreated	TSG-6 expression/ infiltration analysis
B (location 2)	F	IIB	Untreated	TSG-6 expression/ infiltration analysis
D	M	I	Untreated	Infiltration analysis
E	F	IIB	Untreated	Infiltration analysis
F	F	III	Untreated	Infiltration analysis
G	M	IIB	Untreated	TSG-6 expression/ infiltration analysis
J	M	III	Untreated	TSG-6 expression/ infiltration analysis
K	F	III	Untreated	TSG-6 expression/ infiltration analysis
1	M	IIB	Unknown	TSG-6 expression/ infiltration analysis
2	F	IIB	Unknown	TSG-6 expression/ infiltration analysis
3	M	Unknown	Unknown	Infiltration analysis
4	M	IIB	Unknown	TSG-6 expression/ infiltration analysis

Melanoma

Sample name	Sex	Clinical staging	Treatment at time of resection	Analysis performed
2567	M	IV	Naïve	TSG-6 expression
3149	F	IV	Naïve	TSG-6 expression
3185	M	IV	Naïve	TSG-6 expression
3636	F	IV	Naïve	TSG-6 expression
4069	M	III	Naïve	TSG-6 expression
2397	M	IV	Naïve	TSG-6 expression
2706	M	IV	Naïve	TSG-6 expression
3025	F	IV	Naïve	TSG-6 expression

Supplementary Table 1: Summary of patient characteristics used for multi-immunofluorescence depicted in Figure. 2 and Figure. 4. Pathological staging is represented with stage of Tumor (T), Lymph Node (N) and Metastasis (M).

Supplementary Table 2

Antibodies for murine CyTOF	Source	Identifier	Dilution
Anti-CD45 (clone 30-F11)	Standard BioTools	Cat# 3089005B	1:200
Anti-I-A/I-E (MHCII) (clone M5/114.15.2)	Biolegend	Cat# 107637	1:800
Anti-CD4 (clone RMP4-5)	Biolegend	Cat# 100506	1:200
Anti-CD64 (clone X54-5/7.1)	Biolegend	Cat# 139302	1:50
Anti-Ly6G (clone RPA-T4)	Biolegend	Cat# 127620	1:200
Anti-Tbet (clone 4B10)	Biolegend	Cat# 644805	1:75
Anti-VISTA (clone MIH63)	Biolegend	Cat# 150202	1:200
Anti-TNFa (clone MP6-XT22)	Standard BioTools	Cat# 3162002B	1:50
Anti-PD-L2 (clone Ty25)	Biolegend	Cat# 107202	1:100
Anti-CD103 (clone 2E7)	Biolegend	Cat# 121402	1:200
Anti-IFNG (clone XMG1.2)	Biolegend	Cat# 505802	1:25
Anti-Eomes (clone Dan11mag)	eBioscience	Cat# 14-4875-82	1:50
Anti-CD80 (clone 16-10A1)	Biolegend	Cat# 104710	1:800
Anti-CD163 (clone EPR19518)	Abcam	Cat# ab182422	1:50
Anti-GATA3 (clone TWAJ)	eBioscience	Cat# 14-9966-82	1:100
Anti-ICOS (clone 7E.17G9)	eBioscience	Cat# 14-9942-85	1:100
Anti-F4/80 (clone BM8)	Biolegend	Cat# 123143	1:100
Anti-CD86 (clone GL-1)	Biolegend	Cat# 105002	1:100
Anti-GranzymeB (clone QA16A02)	Biolegend	Cat# 372202	1:100
Anti-CD115 (clone AFS98)	Standard BioTools	Cat# 3144012B	1:50
Anti-FoxP3 (clone FJK-16s)	Standard BioTools	Cat# 3158003A	1:100
Anti- CD8a (clone 53-6.7)	Standard BioTools	Cat# 3146003B	1:200
Anti- CD19 (clone 6D5)	Standard BioTools	Cat# 3149002B	1:200
Anti-Ly6C (clone HK1.4)	Standard BioTools	Cat# 3150010B	1:400
Anti-CD25 (clone 3C7)	Standard BioTools	Cat# 3151007B	1:50
Anti-CD3e (clone 145-2C11)	Standard BioTools	Cat# 3152004B	1:100
Anti-CTLA-4 (clone UC10-4B9)	Standard BioTools	Cat# 3154008B	1:100
Anti-LAG3 (clone C9B7W)	Standard BioTools	Cat# 3174019B	1:100
Anti- iNOS (clone CXNFT)	Standard BioTools	Cat# 3161011B	1:100

Anti-PD-1 (clone J43)	Standard BioTools	Cat# 3159023B	1:100
Anti-CD206 (clone C068C2)	Standard BioTools	Cat# 3169021B	1:200
Anti-NK1.1 (clone PK136)	Standard BioTools	Cat# 3170002B	1:100
Anti-CD11b (clone M1/70)	Standard BioTools	Cat# 3172012B	1:1600
Anti-Arginase1 (clone 8C9)	Santacruz	Cat# sc-47715	1:400
Anti-CD11c (clone N418)	Standard BioTools	Cat# 3153016B	1:50
Anti-PD-L1 (clone 10F.9G2)	Standard BioTools	Cat# 3142003B	1:200

Supplementary Table 2: List of antibodies used for CyTOF analysis.

References:

1 Peng, J. *et al.* Single-cell RNA-seq highlights intra-tumoral heterogeneity and malignant progression in pancreatic ductal adenocarcinoma. *Cell Res* **29**, 725-738, doi:10.1038/s41422-019-0195-y (2019).

2 Elyada, E. *et al.* Cross-Species Single-Cell Analysis of Pancreatic Ductal Adenocarcinoma Reveals Antigen-Presenting Cancer-Associated Fibroblasts. *Cancer Discov* **9**, 1102-1123, doi:10.1158/2159-8290.CD-19-0094 (2019).
