# Supplementary material for: TSG-6+ cancer-associated fibroblasts modulate myeloid cell responses and impair anti-tumor response to immune checkpoint therapy in pancreatic cancer
Source: Nat Commun. 2024 Jul 10;15:5291. doi: 10.1038/s41467-024-49189-x (PMC11237123; doi:10.1038/s41467-024-49189-x)
Supplement: Supplementary file 3 — Description of Additional Supplementary Files [file 41467_2024_49189_MOESM3_ESM.pdf]

Description of Additional Supplementary Files

File Name: Supplementary Data 1
Description: Top 20 genes for each cluster identified from our murine scRNAseq datasets (CD45 positive and CD45 negative).
