# Supplementary material for: TSG-6+ cancer-associated fibroblasts modulate myeloid cell responses and impair anti-tumor response to immune checkpoint therapy in pancreatic cancer
Source: Nat Commun. 2024 Jul 10;15:5291. doi: 10.1038/s41467-024-49189-x (PMC11237123; doi:10.1038/s41467-024-49189-x)
Supplement: Supplementary file 5 — Reporting Summary [file 41467_2024_49189_MOESM5_ESM.pdf]

nature portfolio

Corresponding author(s): Padmanee Sharma
Last updated by author(s): May 16, 2024

Reporting Summary

Nature Portfolio wishes to improve the reproducibility of the work that we publish. This form provides structure for consistency and transparency in reporting. For further information on Nature Portfolio policies, see our Editorial Policies and the Editorial Policy Checklist.

Statistics

For all statistical analyses, confirm that the following items are present in the figure legend, table legend, main text, or Methods section.

n/a	Confirmed	
☐	☒	The exact sample size (n) for each experimental group/condition, given as a discrete number and unit of measurement
☐	☒	A statement on whether measurements were taken from distinct samples or whether the same sample was measured repeatedly
☐	☒	The statistical test(s) used AND whether they are one- or two-sided<br>*Only common tests should be described solely by name; describe more complex techniques in the Methods section.*
☐	☒	A description of all covariates tested
☐	☒	A description of any assumptions or corrections, such as tests of normality and adjustment for multiple comparisons
☐	☒	A full description of the statistical parameters including central tendency (e.g. means) or other basic estimates (e.g. regression coefficient) AND variation (e.g. standard deviation) or associated estimates of uncertainty (e.g. confidence intervals)
☐	☒	For null hypothesis testing, the test statistic (e.g. F, t, r) with confidence intervals, effect sizes, degrees of freedom and P value noted<br>*Give P values as exact values whenever suitable.*
☒	☐	For Bayesian analysis, information on the choice of priors and Markov chain Monte Carlo settings
☐	☒	For hierarchical and complex designs, identification of the appropriate level for tests and full reporting of outcomes
☐	☒	Estimates of effect sizes (e.g. Cohen's d, Pearson's r), indicating how they were calculated

Our web collection on statistics for biologists contains articles on many of the points above.

Software and code

Policy information about availability of computer code

Data collection	Seurat V4.3.0, Helios 6.5.358, Inform (Akoya)
Data analysis	R software 4.2.3 (packages flowCore, CATLAYST, cytofkit, FlowSOM) , Loupe Browser v7.0, Cellranger v3.0.2, HALO v4.04, Prism v8.0 and v9.0, MSigDB v7.1

For manuscripts utilizing custom algorithms or software that are central to the research but not yet described in published literature, software must be made available to editors and reviewers. We strongly encourage code deposition in a community repository (e.g. GitHub). See the Nature Portfolio guidelines for submitting code & software for further information.

Data

Policy information about availability of data

All manuscripts must include a data availability statement. This statement should provide the following information, where applicable:

- Accession codes, unique identifiers, or web links for publicly available datasets
- A description of any restrictions on data availability
- For clinical datasets or third party data, please ensure that the statement adheres to our policy

The scRNAseq datasets generated for this study have been deposited in NCBI Sequence Read Archive (SRA) repository under the BioProject accession number PRJNA1099275. All requests for the data should be made to the corresponding author (P. S.), following verification of any intellectual property or confidentiality obligations. The human PDAC scRNAseq publicly available data used in this study are available in the Genome Sequence Archive under the accession code CRA001160 and project PRJCA001063. Human GRCh38 genome is available under the accession code GCF_000001405.26.The remaining data are available within

the Article, Supplementary Information or Source Data file.

Research involving human participants, their data, or biological material

Policy information about studies with human participants or human data. See also policy information about sex, gender (identity/presentation), and sexual orientation and race, ethnicity and racism.

Reporting on sex and gender	Patient related information including sex and gender have been included in the patient characteristics in Supplementary Table 1. Gender of participants was determined based on self reporting. Since the study involves the understanding of cancer-associated fibroblast mediators in ICT responses, sex- and gender-based analyses were not performed.
Reporting on race, ethnicity, or other socially relevant groupings	Patient related information including race, ethnicity, or other socially relevant groupings is not reported.
Population characteristics	Patient characteristics has been described in Supplementary Table 1.
Recruitment	Patients were consented under the IRB approved protocol PA13-0291 before surgery or sample collection.
Ethics oversight	Patient samples were collected after appropriate informed consent was obtained on MD Anderson internal review board-approved protocol no. PA13-0291. No financial compensation was provided for participation in the trial protocol. All patients signed informed consents for participation in PA13-0291 before surgery or sample collection. The clinical characteristics of individual patients are shown in Supplementary Table 1.

Note that full information on the approval of the study protocol must also be provided in the manuscript.

Field-specific reporting

Please select the one below that is the best fit for your research. If you are not sure, read the appropriate sections before making your selection.

☒ Life sciences ☐ Behavioural & social sciences ☐ Ecological, evolutionary & environmental sciences

For a reference copy of the document with all sections, see nature.com/documents/nr-reporting-summary-flat.pdf

Life sciences study design

All studies must disclose on these points even when the disclosure is negative.

Sample size	Sample sizes were chosen based on availability of patients. For murine experiments, no statistical method was used to predetermine the sample size, however, the sizes used were based on established statistical methods (PMID: 29386977).
Data exclusions	No data were excluded
Replication	Replication has been described in the method section as well in the figure legends wherever involved
Randomization	Post tumor injection, the mice were assessed for initial implantation and then randomly segregated into groups to maintain a common median tumor size prior to treatment. No randomization was performed for human studies as only baseline tissues were assessed.
Blinding	The tumor survival and treatment for the murine experiments were performed by individuals who were blinded to the study and outcome. For human studies, the investigators were blinded during data collection.

Reporting for specific materials, systems and methods

We require information from authors about some types of materials, experimental systems and methods used in many studies. Here, indicate whether each material, system or method listed is relevant to your study. If you are not sure if a list item applies to your research, read the appropriate section before selecting a response.

Materials & experimental systems

n/a	Involved in the study	
☐	☒	Antibodies
☐	☒	Eukaryotic cell lines
☒	☐	Palaeontology and archaeology
☐	☒	Animals and other organisms
☒	☐	Clinical data
☒	☐	Dual use research of concern
☒	☐	Plants

Methods

n/a	Involved in the study	
☒	☐	ChIP-seq
☒	☐	Flow cytometry
☒	☐	MRI-based neuroimaging

Antibodies

Antibodies used

1) In vivo antibodies used: Anti-CTLA-4 (3 doses- 200, 100, 100ug/ mouse) (clone 9H10, cat# BP1064) and anti-PD-1 (3 doses- 250ug/ mouse) (clone RMP1-14, cat# BP0146) antibodies were purchased from BioXcell (West Lebanon,NH). Anti-TSG-6 antibody (50ug/ mouse) (cat #MAB2104) was purchased from R&D systems (Minneapolis, MN).
2) The antibodies used for human immunofluorescence are: CD68 (Dako-Agilent, clone PGM-1, 1:25 dilution), CD163 (Leica Biosystems, clone 10D6, 1:50 dilution), CD44 (Cell Signaling Technologies, clone E7K27, 1:800 Dilution), TSG-6 (Novusbio, clone 38637, 1:40 dilution), and PanCK (Dako-Agilent, clone AE1/3, 1:500 dilution).
3) Antibodies for immunohistochemistry: anti-alpha SMA antibody (Abcam, clone ab5694) at 1:1000 dilution.
4) The antibodies used for murine immunofluorescence are: CD44 (Cell Signaling Technologies, clone CST37279, 1:300 dilution), CD68 (Abcam, clone ab125212, 1:200 dilution).
5) The antibodies used for CyTOF have been described in Supplementary Table 2 and below:
Antibodies Source Identifier Dilution
Anti-CD45 (clone 30-F11) Standard BioTools Cat# 3089005B 1:200
Anti-I-A/I-E (MHCII) (clone M5/114.15.2) Biolegend Cat# 107637 1:800
Anti-CD4 (clone RMP4-5) Biolegend Cat# 100506 1:200
Anti-CD64 (clone X54-5/7.1) Biolegend Cat# 139302 1:50
Anti-Ly6G (clone RPA-T4) Biolegend Cat# 127620 1:200
Anti-Tbet (clone 4B10) Biolegend Cat# 644805 1:75
Anti-VISTA (clone MIH63) Biolegend Cat# 150202 1:200
Anti-TNFa (clone MP6-XT22) Standard BioTools Cat# 3162002B 1:50
Anti-PD-L2 (clone Ty25) Biolegend Cat# 107202 1:100
Anti-CD103 (clone 2E7) Biolegend Cat# 121402 1:200
Anti-IFNG (clone XMG1.2) Biolegend Cat# 505802 1:25
Anti-Eomes (clone Dan11mag) eBioscience Cat# 14-4875-82 1:50
Anti-CD80 (clone 16-10A1) Biolegend Cat# 104710 1:800
Anti-CD163 (clone EPR19518) Abcam Cat# ab182422 1:50
Anti-GATA3 (clone TWAJ) eBioscience Cat# 14-9966-82 1:100
Anti-ICOS (clone 7E.17G9) eBioscience Cat# 14-9942-85 1:100
Anti-F4/80 (clone BM8) Biolegend Cat# 123143 1:100
Anti-CD86 (clone GL-1) Biolegend Cat# 105002 1:100
Anti-GranzymeB (clone QA16A02) Biolegend Cat# 372202 1:100
Anti-CD115 (clone AFS98) Standard BioTools Cat# 3144012B 1:50
Anti-FoxP3 (clone FJK-16s) Standard BioTools Cat# 3158003A 1:100
Anti- CD8a (clone 53-6.7) Standard BioTools Cat# 3146003B 1:200
Anti- CD19 (clone 6D5) Standard BioTools Cat# 3149002B 1:200
Anti-Ly6C (clone HK1.4) Standard BioTools Cat# 3150010B 1:400
Anti-CD25 (clone 3C7) Standard BioTools Cat# 3151007B 1:50
Anti-CD3e (clone 145-2C11) Standard BioTools Cat# 3152004B 1:100
Anti-CTLA-4 (clone UC10-4B9) Standard BioTools Cat# 3154008B 1:100
Anti-LAG3 (clone C9B7W) Standard BioTools Cat# 3174019B 1:100
Anti- iNOS (clone CXNFT) Standard BioTools Cat# 3161011B 1:100
Anti-PD-1 (clone J43) Standard BioTools Cat# 3159023B 1:100
Anti-CD206 (clone C068C2) Standard BioTools Cat# 3169021B 1:200
Anti-NK1.1 (clone PK136) Standard BioTools Cat# 3170002B 1:100
Anti-CD11b (clone M1/70) Standard BioTools Cat# 3172012B 1:1600
Anti-Arginase1 (clone 8C9) Santacruz Cat# sc-47715 1:400
Anti-CD11c (clone N418) Standard BioTools Cat# 3153016B 1:50
Anti-PD-L1 (clone 10F.9G2) Standard BioTools Cat# 3142003B 1:200

Validation

Prevalidated antibodies were purchased based on the manufacturer's description and protocol. For in-house conjugated antibodies for CyTOF, validation was performed on pre-tested cells using the manufacturer's protocol. The details are provided below:

Anti-Mouse CD45, Standard BioTools, 3089005B, https://store.standardbio.com/Cytometry/ConsumablesandReagentsCytometry/MaxparAntibodies/Anti-Mouse%20CD45%20-30-F11-89Y%E2%80%94100%20Tests.

Purified anti-mouse I-A/I-E antibody, Biolegend, 107637, https://www.biolegend.com/en-us/products/purified-anti-mouse-i-a-i-emaxpar-ready-antibody-10095.

Anti-Mouse CD11c, Standard BioTools, 3142003B, https://store.standardbio.com/Cytometry/ConsumablesandReagentsCytometry/MaxparAntibodies/Anti-Mouse%20CD11c%20-N418-142Nd%E2%80%94100%20Tests?cclcl=en_US.

Purified anti-mouse CD4 Antibody, Biolegend, 100505, https://www.biolegend.com/en-us/products/purified-anti-mouse-cd4antibody-484.

Anti-Mouse CD115, Standard BioTools, 3144012B, https://store.standardbio.com/Cytometry/ConsumablesandReagentsCytometry/MaxparAntibodies/Anti-Mouse%20CD115%20-AFS98-144Nd%E2%80%94100%20Tests?cclcl=en_US.

Anti-Mouse CD8a, Standard BioTools, 3146003B, https://store.standardbio.com/Cytometry/ConsumablesandReagentsCytometry/MaxparAntibodies/Anti-Mouse%20CD8a%20-53-6-7-146Nd%E2%80%94100%20Tests?cclcl=en_US.

Purified anti-mouse Ly-6G Antibody, Biolegend, 127637, https://www.biolegend.com/en-us/products/purified-anti-mouse-ly-6gmaxpar-ready-antibody-10114.

Purified anti-mouse/human CD11b Antibody, Biolegend, 101249, https://www.biolegend.com/en-us/products/purified-anti-mousehuman-cd11b-maxpar-ready-antibody-9159.

Anti-Mouse CD19, Standard BioTools, 3149002B, https://store.standardbio.com/Cytometry/ConsumablesandReagentsCytometry/MaxparAntibodies/Anti-Mouse%20CD19%20-6D5-149Sm%E2%80%94100%20Tests?cclcl=en_US.

Purified anti-mouse Ly-6C Antibody, Biolegend, 128039, https://www.biolegend.com/en-us/products/purified-anti-mouse-ly-6cmaxpar-ready-antibody-10115.

Anti-Mouse CD25, Standard BioTools, 3151007B, https://store.standardbio.com/Cytometry/ConsumablesandReagentsCytometry/MaxparAntibodies/Anti-Mouse%20CD25%20-3C7-151Eu%E2%80%94100%20Tests?cclcl=en_US.

Anti-Mouse CD3e, Standard BioTools, 3152004B, https://store.standardbio.com/Cytometry/ConsumablesandReagentsCytometry/MaxparAntibodies/Anti-Mouse%20CD3e%20-145-2C11-152Sm%E2%80%94100%20Tests?cclcl=en_US.

Anti-Mouse CD274/PD-L1, Standard BioTools, 3153031B, https://store.standardbio.com/ccrz__ProductDetails?sku=3153031B&cclcl=en_US.

Anti-Mouse CD152/CTLA-4, Standard BioTools, 3154008B, https://store.standardbio.com/Cytometry/ConsumablesandReagentsCytometry/ MaxparAntibodies/Anti-Mouse%20CD152-CTLA-4%20-UC10-4B9-154Sm%E2%80%94100%20Tests?cclcl=en_US.

Purified anti-T-bet Antibody, Biolegend, 644825, https://www.biolegend.com/en-us/products/purified-anti-t-bet-maxpar-readyantibody-10169.

Purified anti-human/mouse Granzyme B Recombinant Antibody, Biolegend, 372202, https://www.biolegend.com/en-us/products/purified-anti-human-mouse-granzyme-b-recombinant-antibody-14428.

Anti-Mouse Foxp3, Standard BioTools, 3158003A, https://store.standardbio.com/Cytometry/ConsumablesandReagentsCytometry/MaxparAntibodies/Anti-Mouse%20Foxp3%20-FJK-16s-158Gd%E2%80%9450%20Tests?cclcl=en_US.

Anti-Mouse CD279/PD-1, Standard BioTools, 3159023B, https://store.standardbio.com/Cytometry/ConsumablesandReagentsCytometry/ MaxparAntibodies/Anti-Mouse%20CD279-PD-1%20-J43-159Tb%E2%80%94100%20Tests.

Anti-Mouse iNOS, Standard BioTools, 3161011B, https://store.standardbio.com/Cytometry/ConsumablesandReagentsCytometry/MaxparAntibodies/Anti-Mouse%20iNOS%20-CXNFT-161Dy%E2%80%94100%20Tests?cclcl=en_US.

Purified anti-mouse CD273 (B7-DC, PD-L2) Antibody, Biolegend, 107202, https://www.biolegend.com/en-us/products/purified-antimouse-cd273-b7-dc-pd-l2-antibody-2545.

Anti-Mouse LAP/TGFb, Standard BioTools, 3164014B, https://store.standardbio.com/Cytometry/ConsumablesandReagentsCytometry/ MaxparAntibodies/Anti-Mouse%20LAP-TGFb%20-TW7-16B4-164Dy%E2%80%94100%20Tests?cclcl=en_US.

Purified anti-mouse IFN-γ Antibody, Biolegend, 505801, https://www.biolegend.com/en-us/products/purified-anti-mouse-ifngamma-antibody-998.

EOMES Monoclonal Antibody, eBioscience, 14-4875-82, https://www.thermofisher.com/antibody/product/EOMES-Antibody-cloneDan11mag-Monoclonal/14-4875-82.

LEAF™ Purified anti-mouse CD80 Antibody, Biolegend, 104710,https://www.citeab.com/antibodies/518533-104710-leaf-purifiedanti-mouse-cd80-antibody.

ARG1/Arginase 1 Antibody, Santa Cruz Biotechnology, sc- 47715, https://www.scbt.com/p/arginase-1-antibody-8c9.

Purified anti-mouse CD206 (MMR) Antibody, Biolegend, 141701, https://www.biolegend.com/en-us/products/purified-anti-mousecd206-mmr-antibody-7317.

Anti-Mouse NK1.1, Standard BioTools, 3170002B, https://store.standardbio.com/Cytometry/ConsumablesandReagentsCytometry/MaxparAntibodies/Anti-Mouse%20NK1-1%20-PK136-170Er%E2%80%94100%20Tests?cclcl=en_US.

Anti-Human/Mouse CD44, Standard BioTools, 3171003B, https://store.standardbio.com/Cytometry/ConsumablesandReagentsCytometry/ MaxparAntibodies/Anti-Human-Mouse%20CD44%20-IM7-171Yb%E2%80%94100%20Tests?cclcl=en_US.

Gata-3 Monoclonal Antibody, eBioscience, 14-9966-82, https://www.thermofisher.com/antibody/product/Gata-3-Antibody-cloneTWAJ-Monoclonal/14-9966-82.

F4/80 Monoclonal Antibody, eBioscience, 14-4801-82, https://www.thermofisher.com/antibody/product/F4-80-Antibody-cloneBM8-Monoclonal/14-4801-82.

Purified anti-mouse CD86 Antibody, Biolegend, 105002, https://www.biolegend.com/en-us/products/purified-anti-mouse-cd86antibody-257.

CD278 (ICOS) Monoclonal Antibody, eBioscience, 14-9948-82, https://www.thermofisher.com/antibody/product/CD278-ICOSAntibody-clone-ISA-3-Monoclonal/14-9948-82.

Purified anti-mouse CD103 Antibody, Biolegend, 121402, https://www.biolegend.com/en-us/products/purified-anti-mouse-cd103antibody-3572.

Anti-CD64 (clone X54-5/7.1),Biolegend, 139302, https://www.biolegend.com/de-at/products/purified-anti-mouse-cd64-fcgammari-antibody-6690

Anti-VISTA (clone MIH63) Biolegend, 150202, https://www.biolegend.com/en-ie/products/purified-anti-mouse-vista-pd-1h-antibody-11983

Anti-TNFa (clone MP6-XT22), Standard BioTools, 3162002B, https://store.standardbio.com/product_detail/guest-catalog/3162002C

Anti-CD163 (clone EPR19518), Abcam, ab182422, https://www.abcam.com/products/primary-antibodies/cd163-antibody-epr19518-ab182422.html

Anti-Ly6C (clone HK1.4), Standard BioTools, 3150010B, https://store.standardbio.com/product_detail/guest-catalog/3150010B

Eukaryotic cell lines

Policy information about cell lines and Sex and Gender in Research

Cell line source(s)	mT4 pancreatic cell line was generous gift from Dr. David A. Tuveson (Cold Spring Harbor Laboratory, NY). mT4-LS cell line was generated and generously gifted by Dr. Michael Curran (The University of Texas MD Anderson Cancer Center, Houston, TX). B16F10 melanoma cell line was obtained from Dr. I. Fidler (The University of Texas MD Anderson Cancer Center, Houston, TX).
Authentication	We did not authenticate the cell lines.
Mycoplasma contamination	All the cell lines were negative for mycoplasma contamination.
Commonly misidentified lines (See ICLAC register)	No misidentified cell lines were used.

Animals and other research organisms

Policy information about studies involving animals; ARRIVE guidelines recommended for reporting animal research, and Sex and Gender in Research

Laboratory animals	C57BL/6 (5–7 weeks) mice were purchased from the National Cancer Institute (Frederick, MD). Male mice were used for mT4 tumors and female mice were used for B16F10 tumors. All mice were kept in specific pathogen-free conditions in the Animal Resource Center at The University of Texas MD Anderson Cancer Center. The mice were maintained at 20–26 °C, 30–70% humidity and under a 12/12 h light/dark cycle.
Wild animals	This study did not involve any wild animals.
Reporting on sex	We did not perform any sex based studies. The patients used for scRNAseq analysis and immunofluorescence included both male and female patients, however the sample size was not big enough to perform statistically significant analyses between sexes.
Field-collected samples	No field collected samples were used in the study.
Ethics oversight	This research complies with all relevant ethical regulations. The clinical protocol was approved by the internal review board at The University of Texas MD Anderson Cancer Center. All animal experiments were conducted according to protocols approved by the Animal Resource Center at The University of Texas MD Anderson Cancer Center.

Note that full information on the approval of the study protocol must also be provided in the manuscript.
